# Early Escalation to Vancomycin in Severe Pediatric Pneumonia With Pleural Effusion: A Case Report

**DOI:** 10.7759/cureus.79218

**Published:** 2025-02-18

**Authors:** Muhammad Faraaz Ismail, Osama Elassy

**Affiliations:** 1 General Practice, Thumbay University Hospital, Ajman, ARE; 2 Center for Pediatrics and Neonatology, Thumbay University Hospital, Ajman, ARE

**Keywords:** community acquired pneumonia, crp trends, left sided pleural effusion, pediatric pneumonia, vancomycin trough level

## Abstract

Community-acquired pneumonia (CAP) is a significant cause of pediatric morbidity and mortality. While beta-lactams and macrolides are standard treatments, severe cases caused by multidrug-resistant pathogens, such as methicillin-resistant *Staphylococcus aureus* (MRSA), necessitate early escalation to vancomycin. This report emphasizes the importance of clinical judgment, early identification of resistance, and timely intervention in the management of severe pediatric pneumonia.

A two-year-old female presented with fever, cough, and progressive respiratory symptoms. Chest X-ray revealed left lung consolidation, bilateral opacities, and blunting of the left costophrenic angle. Blood cultures were negative, and sputum cultures could not be obtained. Initial therapy with ceftriaxone and clarithromycin failed to achieve clinical improvement. Vancomycin was initiated and monitored with therapeutic trough levels. The patient showed significant clinical improvement with vancomycin, evidenced by CRP trends and resolution of pleural effusion on imaging.

This case highlights the importance of early identification of antibiotic resistance, therapeutic drug monitoring for vancomycin, and the role of imaging in guiding management decisions. The absence of microbiological confirmation emphasized the critical need for clinical judgment. Vancomycin remains a fundamental therapy for resistant pathogens like MRSA. Timely treatment, imaging, and careful monitoring can significantly improve outcomes in severe pediatric pneumonia.

## Introduction

Community-acquired pneumonia (CAP) is one of the leading causes of hospitalization in children worldwide, particularly in low and middle-income countries. While most cases are treated effectively with beta-lactams or macrolides, multidrug-resistant organisms, such as methicillin-resistant *Staphylococcus aureus* (MRSA), pose significant management challenges [1,2]. Severe CAP can lead to complications such as pleural effusion, empyema, and respiratory failure, necessitating timely recognition and intervention [2].

Vancomycin remains a fundamental therapy for MRSA-related pneumonia and is recommended in cases of severe CAP where first-line antibiotics fail. However, achieving therapeutic efficacy with vancomycin requires careful dosing and monitoring to balance efficacy and safety, as pediatric patients often require higher doses to meet recommended trough levels [3,4]. Studies suggest that maintaining vancomycin trough concentrations between 10 and 15 mg/L optimizes clinical outcomes while minimizing nephrotoxicity risk; however, some institutions may prefer therapeutic concentrations to be maintained between 5 and 10 mg/L [4]. Despite these recommendations, real-world limitations, such as delays in microbiological confirmation or subtherapeutic dosing, can complicate management [3].

This case highlights the successful management of a child with severe bronchopneumonia complicated by pleural effusion who responded to vancomycin therapy, despite the absence of microbiological confirmation. It highlights the importance of early antibiotic escalation, therapeutic monitoring, and the use of imaging and inflammatory markers in guiding clinical decisions.

## Case presentation

Patient information and initial presentation

A two-year-old female presented on 27 January 2025 with a four-day history of fever and persistent cough. On examination, she was febrile but alert, with no significant respiratory distress. Chest auscultation revealed bronchial breathing and bilateral crepitations. Based on these findings, a diagnosis of CAP was suspected, and investigations were initiated.

Investigations and timeline

**Figure 1:**
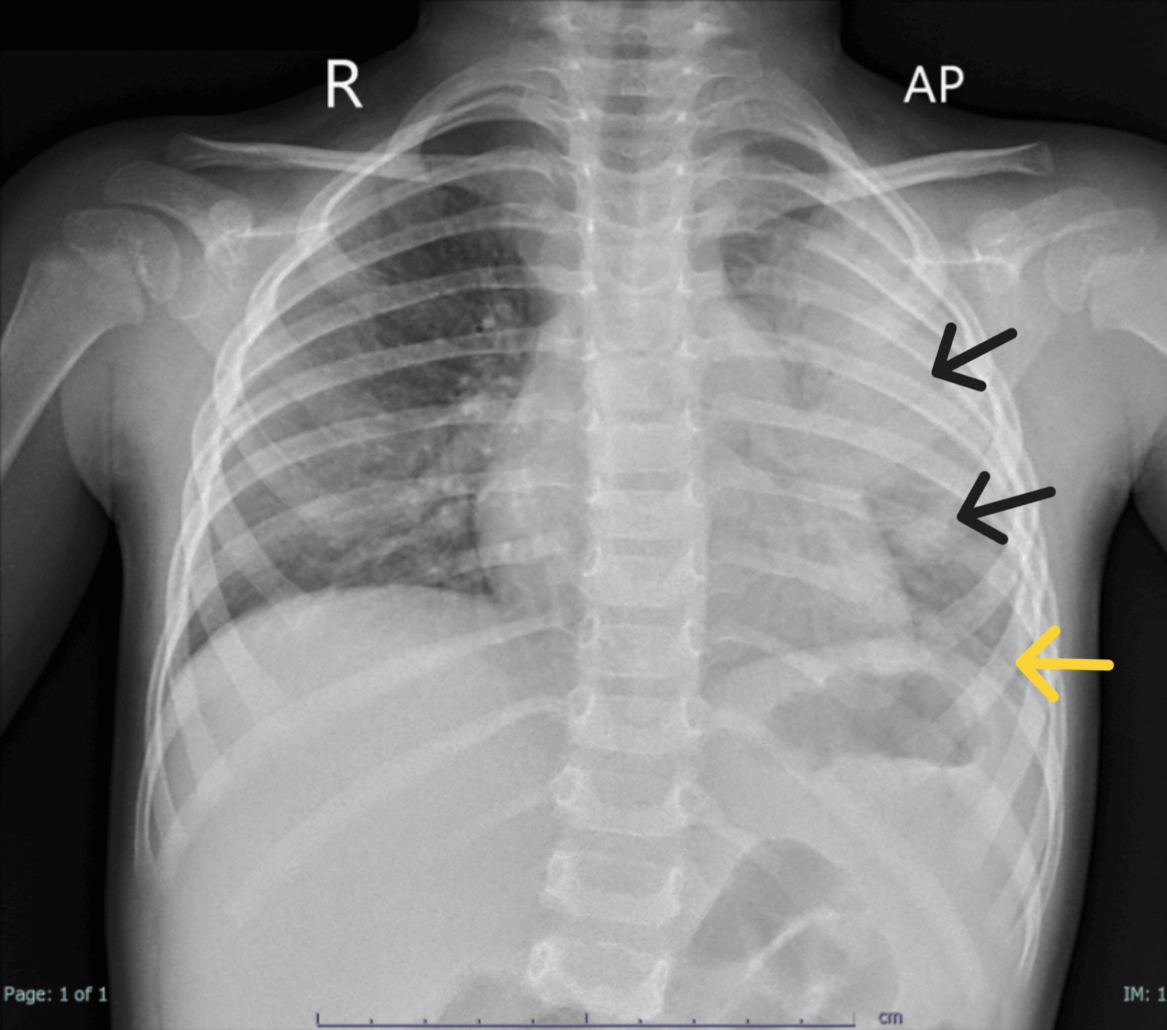
Chest X-ray (erect, AP view) on 27 January 2025 demonstrates left lung consolidation with bilateral reticular nodular opacities Blunting of the left costophrenic angle is observed, suggesting the presence of pleural effusion. The cardiac shadow appears normal, and the trachea and mediastinum are centrally positioned. These findings are consistent with severe CAP complicated by early pleural effusion. Black arrows: Left lung consolidation, suggestive of pneumonia; Yellow arrow: Costophrenic blunting, suggestive of pleural effusion AP: anteroposterior; CAP: community-acquired pneumonia

On 27 January 2025, a chest X-ray (Figure 1) revealed left lung consolidation, bilateral reticular nodular opacities, and blunting of the left costophrenic angle, suggesting pleural effusion. Laboratory results showed a markedly elevated C-reactive protein (CRP) level of 272 mg/L (reference range: <10 mg/L) and a total leukocyte count (TLC) of 18.7 × 10⁹/L (reference range: 5-15 × 10⁹/L) with 78.2% neutrophils (reference range: 30-60%), consistent with significant bacterial infection.

Given these findings, blood and urine cultures were obtained prior to antibiotic initiation. Empiric treatment with ceftriaxone and clarithromycin was started based on standard guidelines for CAP in children.

By 28 January 2025, the CRP level had risen to 320.5 mg/L, indicating worsening infection. Persistent fever and lack of clinical improvement, despite treatment with ceftriaxone and clarithromycin, prompted a reassessment of the underlying pathogen and the need for broader antimicrobial coverage.

Although the patient had no major MRSA risk factors, such as recent hospitalization, prior MRSA infection, immunosuppression, or indwelling medical devices, the failure of first-line antibiotics and the presence of pleural effusion raised suspicion for a resistant bacterial etiology. MRSA has been increasingly recognized as a cause of severe CAP in previously healthy children, particularly in cases with rapid progression and pleural involvement. While local MRSA prevalence data for pediatric CAP were unavailable, the rising incidence of *Staphylococcus aureus *pneumonia in community settings supported the decision to broaden antibiotic coverage.

Given these factors, vancomycin therapy was initiated at a dose of 15 mg/kg every 6 hours to provide coverage for suspected MRSA pneumonia, aligning with current recommendations for empirical treatment in cases of CAP with high suspicion of resistant pathogens.

**Figure 2:**
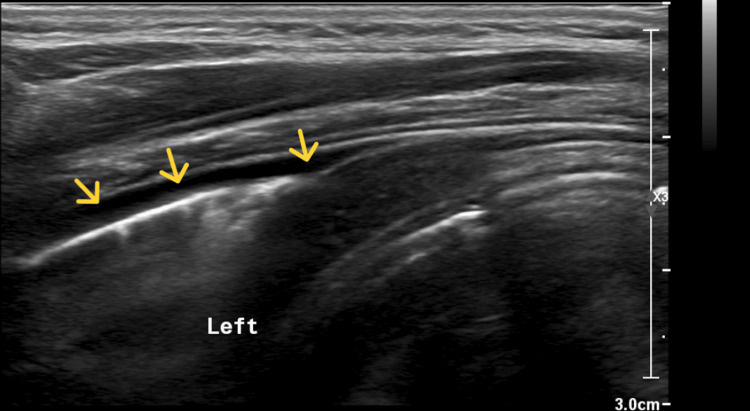
Ultrasound of the left chest wall on 29 January 2025 revealed minimal pleural effusion (<50 mL) on the left side No abnormalities were noted in the surrounding chest wall soft tissues, and the diaphragm exhibited normal movements. These findings confirmed the presence of pleural effusion identified on the initial chest X-ray. Yellow arrows: Anechoic fluid collection, suggestive of small pleural effusion

On 29 January 2025, a chest ultrasound (Figure 2) confirmed minimal left-sided pleural effusion (<50 mL), with no effusion detected on the right side. Vancomycin trough monitoring revealed a level of 4.29 mg/L, which was below the recommended therapeutic range. However, the CRP level decreased to 166.2 mg/L, indicating partial improvement. Blood cultures, obtained on 27 January 2025, were reported as negative for bacterial growth after 48 hours of incubation.

By 30 January 2025, the vancomycin trough level increased to 5.2 mg/L, within the therapeutic range of 5-10 μg/mL for pediatric infections. The CRP level further declined to 99.9 mg/L, reflecting continued improvement. Urine cultures, obtained on 27 January 2025, were reported as negative for bacterial growth after 72 hours of incubation.

On 31 January 2025, the CRP level decreased further to 57.7 mg/L, nearing normalization. The patient was afebrile and showed significant improvement in respiratory status.

**Figure 3:**
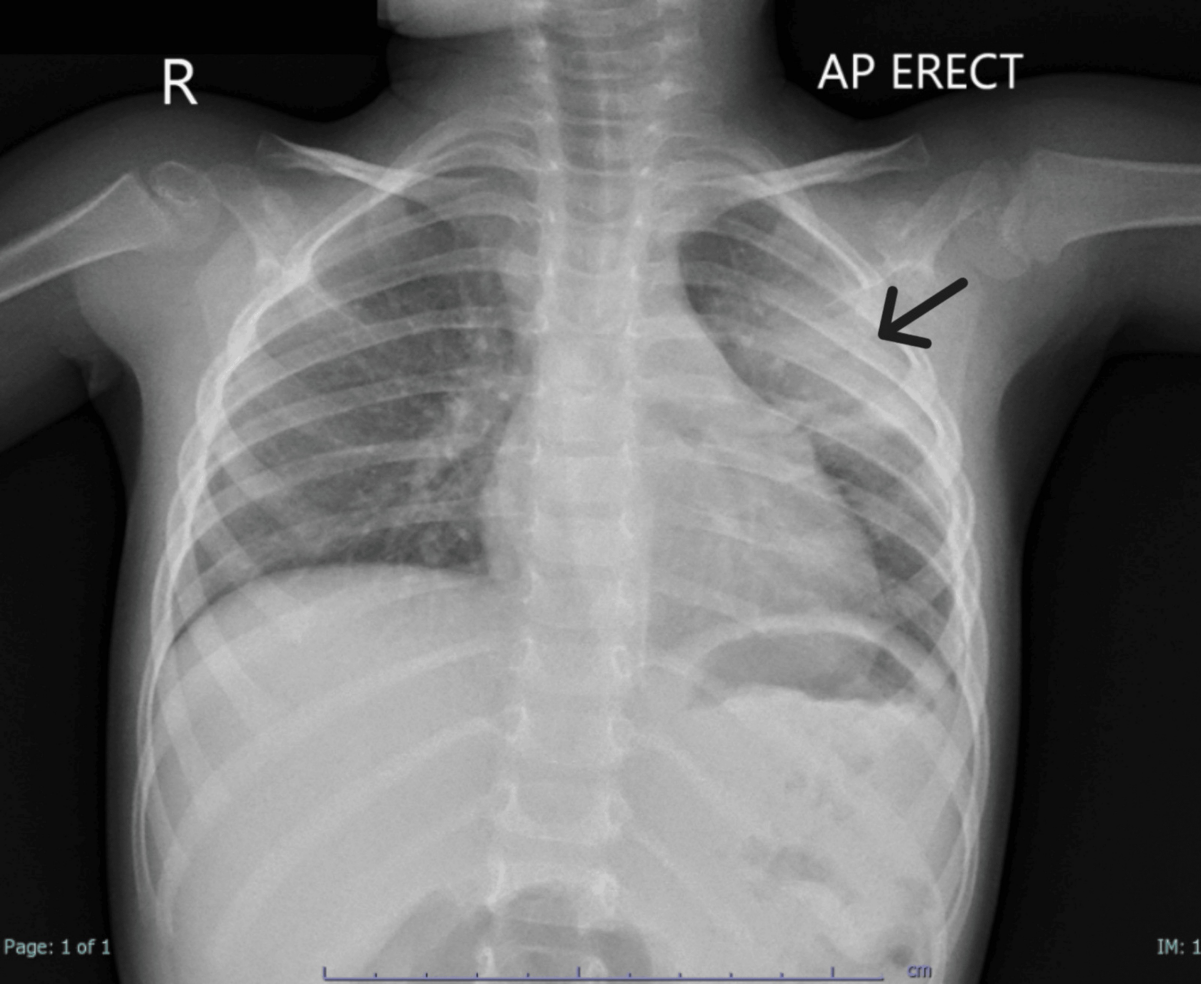
Chest X-ray (erect, AP view) on 01 February 2025 shows significant resolution of the pleural effusion with clear bilateral costophrenic angles A residual heterogeneous opacity is noted in the left upper lobe, consistent with resolving pneumonia. The cardiac shadow remains normal, and the trachea and mediastinum are centrally positioned, reflecting overall clinical and radiological improvement. Black arrow: Residual consolidation in the left upper lobe, suggestive of residual and improving lobar pneumonia AP: anteroposterior

A follow-up chest X-ray performed on 01 February 2025 (Figure 3) demonstrated resolution of the pleural effusion, with clear bilateral costophrenic angles. A residual heterogeneous opacity was noted in the left upper lobe, consistent with resolving pneumonia.

Treatment and follow-up

The child showed marked clinical improvement by Day 4 of vancomycin therapy. She was then discharged on 02 February 2025 with a five-day course of oral Augmentin and scheduled for an outpatient follow-up to monitor and ensure complete recovery. Blood cultures obtained on 27 January 2025 remained negative for bacterial growth after five days of incubation, further supporting the absence of bacteremia.

## Discussion

Diagnostic challenges and role of cultures

The absence of microbiological confirmation posed a significant diagnostic challenge. Blood and urine cultures were negative, and sputum cultures were not obtained due to the inherent difficulty of collecting viable samples in young children [5,6]. Such limitations are well-documented in pediatric pneumonia, where blood cultures have a low sensitivity, often yielding positive results in less than 10% of cases [5]. This highlights the critical role of imaging, inflammatory markers like CRP, and clinical judgment to guide therapy when microbiological data are unavailable. CRP, in particular, has been shown to differentiate bacterial from viral pneumonia with modest sensitivity (70%) and specificity (65%), making it a valuable adjunct in the diagnostic process [6]. These tools are essential in severe cases like this, where timely intervention can prevent complications.

Although the patient had no documented risk factors for MRSA colonization, increasing evidence suggests that MRSA-related CAP can occur in previously healthy children, particularly in cases with rapid clinical deterioration and lung involvement [5]. The lack of clinical improvement, persistent fever, and presence of pleural effusion necessitated a re-evaluation of potential resistant pathogens. This case aligns with literature describing rising MRSA pneumonia cases in pediatric patients, where early broad-spectrum antibiotic coverage is warranted in high-risk scenarios [3-6]. Although local MRSA prevalence data were not available, the decision to initiate vancomycin was guided by clinical experience, imaging findings, and treatment failure with first-line antibiotics. This approach aligns with current recommendations for suspected MRSA pneumonia and reflects the best clinical judgment in the absence of definitive microbiological confirmation.

Therapeutic drug monitoring (TDM) for vancomycin

Vancomycin trough levels were carefully monitored during treatment, achieving 5.2 mg/L on 30 January 2025, within the therapeutic range of 5-10 mg/L recommended for pediatric infections [4]. Maintaining therapeutic levels is crucial to ensure treatment efficacy while minimizing the risk of nephrotoxicity, particularly in young children who have higher drug clearance rates compared to adults [4]. Clinical improvement in this case emphasizes the importance of the timely initiation and careful monitoring of vancomycin in managing severe pneumonia [3,4]. Additionally, early and appropriate antibiotic therapy remains critical in reducing mortality and long-term complications, as delayed treatment has been associated with poorer outcomes [5].

Role of imaging in management

Early imaging was of great importance in detecting pleural effusion and guiding therapy. The initial chest X-ray identified the presence of pleural effusion, which was later confirmed and monitored with ultrasound. Ultrasound has been advocated as a valuable, non-invasive bedside tool for managing pediatric pneumonia, offering diagnostic utility without the risks of radiation exposure [2,5]. However, its limitations in evaluating certain lung regions, such as the right middle lobe, highlight the need for a multimodal imaging approach in complex cases [6]. Serial imaging, including follow-up chest X-rays, corroborated the clinical improvement and demonstrated the resolution of pleural effusion. This case highlights the essential role of imaging in managing severe CAP [2,6].

Antibiotic stewardship and clinical vigilance

This case illustrates the importance of balancing the principles of antibiotic stewardship with the urgency of life-saving treatment. The failure of first-line antibiotics necessitated timely escalation to vancomycin, a fundamental therapy for suspected MRSA pneumonia [1-4]. Early intervention in cases of suspected resistance is crucial to prevent complications such as pleural effusion and sepsis. This case emphasizes the need for clinical vigilance in recognizing resistance and complications, particularly in young children, where delays in appropriate therapy can result in poorer outcomes [2-4].

## Conclusions

This case highlights the successful management of severe bronchopneumonia with pleural effusion in a two-year-old child who failed to respond to first-line antibiotics. The absence of microbiological confirmation emphasizes the importance of clinical judgment, imaging, and inflammatory markers like CRP in guiding therapy. Vancomycin remains a fundamental therapy for resistant pathogens like MRSA, with therapeutic drug monitoring ensuring safe and effective treatment. Timely intervention and structured escalation strategies are critical in preventing delays in life-saving treatment and improving outcomes in severe pediatric pneumonia.
